# 3′,4′-Dichloro­biphenyl-4-yl 2,2,2-trichloro­ethyl sulfate

**DOI:** 10.1107/S1600536810020362

**Published:** 2010-06-09

**Authors:** Xueshu Li, Sean Parkin, Michael W. Duffel, Larry W. Robertson, Hans-Joachim Lehmler

**Affiliations:** a The University of Iowa, Department of Occupational and Environmental Health, UI Research Campus, 124 IREH, Iowa City, IA 52242-5000, USA; b University of Kentucky, Department of Chemistry, Lexington, KY 40506-0055, USA; c Division of Medicinal and Natural Products Chemistry, College of Pharmacy, University of Iowa, Iowa City, IA 52242, USA

## Abstract

The four independent mol­ecules in the asymmetric unit of the title compound, C_14_H_9_Cl_5_O_4_S, are related by pseudo-inversion centres. The mol­ecules have C_aromatic_—O bond lengths ranging from 1.426 (10) to 1.449 (9) Å and biphenyl-4-yl sulfate ester bond lengths ranging from 1.563 (6) to 1.586 (6) Å, which is comparable to structurally related sulfuric acid diesters. The dihedral angles between the benzene rings range from 22.5 (4) to 29.1 (4)° and are significantly smaller than the calculated dihedral angle of 41.2°.

## Related literature

For the structures of similar sulfuric acid biphenyl-4-yl ester 2,2,2-trichloro-ethyl esters, see: Li *et al.* (2008 ▶, 2010*a* ▶,*b* ▶). For a review of the structures of sulfuric acid aryl mono esters, see: Brandao *et al.* (2005 ▶). For further discussion of dihedral angles in chlorinated biphenyl derivatives, see: Lehmler *et al.* (2002 ▶); Shaikh *et al.* (2008 ▶); Vyas *et al.* (2006 ▶). For additional background on polychlorinated biphenyls, see: Letcher *et al.* (2000 ▶); Robertson & Hansen (2001 ▶); Liu *et al.* (2004*a* ▶,*b* ▶); Liu *et al.* (2006 ▶, 2009 ▶); Sacco & James (2005 ▶); Tampal *et al.* (2002 ▶). For software used to caculate dihedral angles, see: Carpenter *et al.* (1980 ▶).
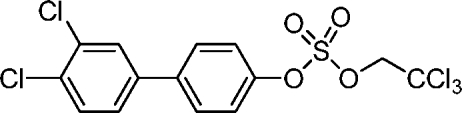


## Experimental

### 

#### Crystal data

C_14_H_9_Cl_5_O_4_S
*M* *_r_* = 450.52
Monoclinic, 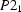

*a* = 7.2491 (1) Å
*b* = 40.5988 (7) Å
*c* = 12.1145 (2) Å
β = 106.1551 (7)°
*V* = 3424.57 (9) Å^3^
*Z* = 8
Mo *K*α radiation
μ = 0.99 mm^−1^
*T* = 90 K
0.40 × 0.34 × 0.18 mm

#### Data collection

Nonius KappaCCD diffractometer
Absorption correction: multi-scan (*SCALEPACK*; Otwinowski & Minor, 1997 ▶) *T*_min_ = 0.658, *T*_max_ = 0.843
44794 measured reflections
14652 independent reflections
8149 reflections with *I* > 2σ(*I*)
*R*_int_ = 0.110

#### Refinement

*R*[*F* ^2^ > 2σ(*F* ^2^)] = 0.062
*wR*(*F* ^2^) = 0.156
*S* = 1.00
14652 reflections
769 parameters
249 restraints
H-atom parameters constrained
Δρ_max_ = 1.11 e Å^−3^
Δρ_min_ = −0.59 e Å^−3^
Absolute structure: Flack (1983 ▶), 6676 Friedel pairs
Flack parameter: 0.10 (9)

### 

Data collection: *COLLECT* (Nonius, 1998 ▶); cell refinement: *SCALEPACK* (Otwinowski & Minor, 1997 ▶); data reduction: *DENZO-SMN* (Otwinowski & Minor, 1997 ▶); program(s) used to solve structure: *SHELXS97* (Sheldrick, 2008 ▶); program(s) used to refine structure: *SHELXL97* (Sheldrick, 2008 ▶); molecular graphics: *XP* in *SHELXTL* (Sheldrick, 2008 ▶); software used to prepare material for publication: *SHELXL97* and local procedures.

## Supplementary Material

Crystal structure: contains datablocks I, global. DOI: 10.1107/S1600536810020362/lh5052sup1.cif

Structure factors: contains datablocks I. DOI: 10.1107/S1600536810020362/lh5052Isup2.hkl

Additional supplementary materials:  crystallographic information; 3D view; checkCIF report

## Figures and Tables

**Fig. 1.**
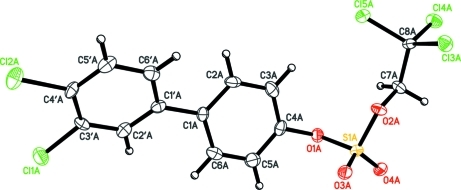
View of one of the four independent molecules of the title compound showing the atom-labeling scheme. Displacement ellipsoids are drawn at the 50% probability level.

## supplementary crystallographic information

### Comment

Hydroxylated polychlorinated biphenyls (PCBs) are an important class of metabolites of PCBs (Letcher *et al.*, 2000) that can be further metabolized to PCB glucuronides (Tampal *et al.*, 2002) or sulfates (Liu *et al.*, 2006; Liu *et al.*, 2009; Sacco & James, 2005). The chemical structure and toxicity of these glucuronide and sulfate metabolites are only poorly investigated, in part because authentic standards are not readily available or because of their limited chemical stability. Here we report the crystal structure of a 2,2,2-trichloroethyl-protected sulfate of 3',4'-dichloro-biphenyl-4-ol, an intermediate of the synthesis of the corresponding sulfate monoester.

The C_Ar_—O (i.e. O1—C4) bond lengths of the title compound are 1.431 (10) Å (O1A—C4A), 1.426 (10) Å (O1B—C4B), 1.427 (10) Å (O1C—C4C) and 1.449 (9) Å (O1D—C4D), respectively. In related sulfuric acid diesters without chlorine substituents in the sulfated phenyl ring, the analogous C_Ar_—O bond lengths were comparable and ranged from 1.426 (2) to 1.435 (5) Å (Li *et al.*, 2010*a*,*b*; Li *et al.*, 2008). A much shorter C_Ar_—O bond length was observed in 2',3,5',5-tetrachloro-biphenyl-4-yl 2,2,2-trichloroethyl sulfate with 1.405 (4) Å (Li *et al.*, 2010*b*). Similar to sulfate monoesters (Brandao *et al.*, 2005), the differences in the C_Ar_—O bond lengths of the sulfate diesters are due to a more positive partial charge on the C4 carbon atom in the presence of chlorine substituents, which results in a shorter C_Ar_—O bond length.

The biphenyl-4-yl sulfate ester (i.e. S1—O1) bond lengths of the title compound were 1.571 (6) Å (S1A—O1A), 1.584 (6) Å (S1B—O1B), 1.586 (6) Å (S1C—O1C) and 1.563 (6) Å (S1D—O1D), respectively. These bond lengths are also comparable to related sulfuric acid diesters (Li *et al.*, 2010*a*,*b*; Li *et al.*, 2008), but shorter compared to 2',3,5',5-tetrachloro-biphenyl-4-yl 2,2,2-trichloroethyl sulfate, a sulfuric acid diester with two chlorine substituents in the sulfated phenyl ring (Li *et al.*, 2010*b*). The differences in the biphenyl-4-yl sulfate ester bond lengths are also a due to the presence or absence of electron withdrawing chlorine substituents, which reduce the electron density on the oxygen atom and contribute to a longer and weaker bond in sulfate mono- and diesters with chlorine substituents in the sulfated phenyl ring (Brandao *et al.*, 2005; Li *et al.*, 2010*b*).

The four molecules in the asymmetyric unit are related by a pseudo-inversion center at (0.75056, 0.50005, 0.62549). Molecules with the A & B atom label suffixes are further related by a pseudo-inversion at (0.23935, 0.50071, 0.37554), while molecules C & D are related by a pseudo-inversion at (1.26176, 0.49939, 0.87544).

The dihedral angle Ar—Ar' between the phenyl rings of a PCB derivative determines its three dimensional structure and, thus, its affinity to cellular targets (Lehmler *et al.*, 2002; Shaikh *et al.*, 2008; Vyas *et al.*, 2006). The solid state dihedral angles between the two phenyl rings of the title compound were 27.2 (4)°, 23.5 (4)°, 29.1 (4)° and 22.5 (4)°, respectively. The corresponding solid state dihedral angles of other sulfate diesters without *ortho* chlorine substituents range from 4.9 to 41.8° (Li *et al.*, 2010a; Li *et al.*, 2008). Typically, the dihedral angles of such sulfate diester derivatives are smaller than the calculated dihedral angle of 41.2° (calculated using semi-empirical SCF-MO calculations with an Austin Model 1 (AM1) Hamiltonian as implemented by the Spartan 02 package [Carpenter *et al.*,  1980]). These deviations from the calculated dihedral angles are likely due to crystal packing effects, which allow the sulfate diester molecule to adopt an energetically unfavorable dihedral angle to maximize intermolecular interactions in the crystal. Overall, the differences between solid state and calculated dihedral angles indicate that the biphenyl moiety of biphenyl-4-yl sulfate ester has considerable conformational freedom in interacting with cellular target molecules.

### Experimental

The title compound was synthesized from 3',4'-dichloro-biphenyl-4-ol by sulfation with 2,2,2-trichloroethyl sulfonyl chloride using 4-dimethylaminopyridine as catalyst (Li *et al.*, 2008; Liu *et al.* 2004*a*,*b*). Crystals suitable for crystal structure analysis were obtained by slowly evaporating a methanolic solution of the title compound.

### Refinement

H atoms were found in difference Fourier maps and subsequently placed in idealized positions with constrained C—H distances of 0.99 Å (CH_2_), and 0.95 Å (C_Ar_H) with *U*_iso_(H) values set to 1.2*U*_eq_ of the attached atom.

### Crystal data

**Table:**

C_14_H_9_Cl_5_O_4_S	*F*(000) = 1808
*M**_r_* = 450.52	*D*_x_ = 1.748 Mg m^−^^3^
Monoclinic, *P*2_1_	Mo *K*α radiation, λ = 0.71073 Å
Hall symbol: P 2yb	Cell parameters from 47065 reflections
*a* = 7.2491 (1) Å	θ = 1.0–27.5°
*b* = 40.5988 (7) Å	µ = 0.99 mm^−^^1^
*c* = 12.1145 (2) Å	*T* = 90 K
β = 106.1551 (7)°	Block, colourless
*V* = 3424.57 (9) Å^3^	0.40 × 0.34 × 0.18 mm
*Z* = 8	

### Data collection

**Table:**

Nonius KappaCCD diffractometer	14652 independent reflections
Radiation source: fine-focus sealed tube	8149 reflections with *I* > 2σ(*I*)
graphite	*R*_int_ = 0.110
Detector resolution: 18 pixels mm^-1^	θ_max_ = 27.5°, θ_min_ = 1.8°
ω scans at fixed χ = 55°	*h* = −9→9
Absorption correction: multi-scan (*SCALEPACK*; Otwinowski & Minor, 1997)	*k* = −52→52
*T*_min_ = 0.658, *T*_max_ = 0.843	*l* = 0→15
44794 measured reflections	

### Refinement

**Table:**

Refinement on *F*^2^	Secondary atom site location: difference Fourier map
Least-squares matrix: full	Hydrogen site location: inferred from neighbouring sites
*R*[*F*^2^ > 2σ(*F*^2^)] = 0.062	H-atom parameters constrained
*wR*(*F*^2^) = 0.156	*w* = 1/[σ^2^(*F*_o_^2^) + (0.0685*P*)^2^] where *P* = (*F*_o_^2^ + 2*F*_c_^2^)/3
*S* = 1.00	(Δ/σ)_max_ = 0.001
14652 reflections	Δρ_max_ = 1.11 e Å^−^^3^
769 parameters	Δρ_min_ = −0.59 e Å^−^^3^
249 restraints	Absolute structure: Flack (1983), 6676 Friedel pairs
Primary atom site location: structure-invariant direct methods	Flack parameter: 0.10 (9)

### Special details

**Table:**

Geometry. All esds (except the esd in the dihedral angle between two l.s. planes) are estimated using the full covariance matrix. The cell esds are taken into account individually in the estimation of esds in distances, angles and torsion angles; correlations between esds in cell parameters are only used when they are defined by crystal symmetry. An approximate (isotropic) treatment of cell esds is used for estimating esds involving l.s. planes.
Refinement. Refinement of *F*^2^ against ALL reflections. The weighted *R*-factor wR and goodness of fit *S* are based on *F*^2^, conventional *R*-factors *R* are based on *F*, with *F* set to zero for negative *F*^2^. The threshold expression of *F*^2^ > 2σ(*F*^2^) is used only for calculating *R*-factors(gt) etc. and is not relevant to the choice of reflections for refinement. *R*-factors based on *F*^2^ are statistically about twice as large as those based on *F*, and *R*- factors based on ALL data will be even larger.There is a pseudo inversion at (0.75070 0.50000 0.62576), but it does seem as if the space group really is P2_1_. This came as a great surprise because there seems to be no obvious reason why this structure would be non-centrosymmetric. All indications are that the crystals themselves are non even inversion twins because the Flack (and Hooft 'y') parameters are both zero within a couple of SUs. Although these SUs are a bit larger than the recommendation suggested by Flack. Further tests with various procedures in PLATON (including ADDSYM) suggest "No Obvious Spacegroup Change Needed/Suggested", but the checkCIF implementation of ADDSYM does suggest "ADDSYM Detects Additional (Pseudo) Symm. Elem··· m", but on inspection the structure does not seem to have any kind of mirror plane. Further, the checkCIF implementation of ADDSYM/MISSYM suggests "Potential lattice centering or halving", but again, on inspection of the model and the diffraction data this does not appear to be the case.

### Fractional atomic coordinates and isotropic or equivalent isotropic displacement parameters (Å^2^)

**Table:**

	*x*	*y*	*z*	*U*_iso_*/*U*_eq_	
S1A	0.0897 (3)	0.41379 (5)	0.3801 (2)	0.0212 (6)	
O1A	0.2072 (8)	0.38089 (14)	0.4093 (5)	0.0201 (14)	
O2A	0.2419 (9)	0.44128 (15)	0.4286 (6)	0.0215 (14)	
O3A	0.0317 (9)	0.41873 (16)	0.2587 (6)	0.0263 (15)	
O4A	−0.0441 (8)	0.41263 (15)	0.4467 (6)	0.0284 (16)	
Cl1A	0.5435 (3)	0.24939 (5)	−0.10621 (17)	0.0304 (5)	
Cl2A	0.8863 (3)	0.28876 (5)	−0.1569 (2)	0.0305 (6)	
Cl3A	0.4320 (3)	0.50544 (5)	0.5117 (2)	0.0244 (6)	
Cl4A	0.5427 (3)	0.47314 (5)	0.7332 (2)	0.0261 (6)	
Cl5A	0.6711 (3)	0.44744 (5)	0.5439 (2)	0.0227 (6)	
C1A	0.4851 (13)	0.3429 (2)	0.1757 (9)	0.0197 (19)	
C2A	0.5790 (11)	0.36492 (19)	0.2620 (7)	0.023 (2)	
H2A	0.7079	0.3711	0.2681	0.027*	
C3A	0.4877 (12)	0.37792 (19)	0.3385 (7)	0.0238 (19)	
H3A	0.5529	0.3928	0.3969	0.029*	
C4A	0.3030 (13)	0.3690 (2)	0.3287 (8)	0.020 (2)	
C5A	0.2038 (12)	0.34682 (18)	0.2459 (7)	0.0267 (19)	
H5A	0.0751	0.3408	0.2410	0.032*	
C6A	0.2961 (11)	0.33403 (19)	0.1724 (7)	0.0230 (18)	
H6A	0.2305	0.3185	0.1165	0.028*	
C7A	0.3002 (12)	0.44536 (19)	0.5532 (8)	0.0217 (10)	
H7A1	0.3292	0.4237	0.5915	0.026*	
H7A2	0.1960	0.4559	0.5788	0.026*	
C8A	0.4790 (12)	0.46710 (18)	0.5830 (8)	0.0180 (10)	
C1'A	0.5824 (11)	0.32855 (19)	0.0935 (8)	0.0194 (10)	
C2'A	0.5326 (11)	0.29847 (19)	0.0421 (6)	0.0220 (10)	
H2'A	0.4350	0.2859	0.0609	0.026*	
C3'A	0.6208 (11)	0.28622 (17)	−0.0356 (6)	0.0191 (9)	
C4'A	0.7679 (10)	0.30432 (19)	−0.0626 (7)	0.0207 (10)	
C5'A	0.8189 (10)	0.33416 (18)	−0.0107 (7)	0.0239 (11)	
H5'A	0.9202	0.3463	−0.0270	0.029*	
C6'A	0.7259 (10)	0.34709 (18)	0.0654 (7)	0.0222 (11)	
H6'A	0.7591	0.3683	0.0981	0.027*	
S1B	0.4062 (3)	0.59117 (5)	0.3690 (2)	0.0230 (6)	
O1B	0.2827 (8)	0.62391 (15)	0.3453 (5)	0.0212 (14)	
O2B	0.2532 (9)	0.56336 (14)	0.3155 (6)	0.0197 (14)	
O3B	0.4676 (9)	0.58420 (16)	0.4878 (6)	0.0292 (16)	
O4B	0.5361 (9)	0.59447 (15)	0.3028 (6)	0.0271 (16)	
Cl1B	−0.0381 (3)	0.73774 (5)	0.90518 (18)	0.0349 (6)	
Cl2B	−0.4422 (3)	0.70796 (5)	0.9015 (2)	0.0340 (6)	
Cl3B	0.0707 (3)	0.49999 (5)	0.2225 (2)	0.0258 (6)	
Cl4B	−0.0582 (4)	0.53617 (6)	0.0088 (2)	0.0300 (6)	
Cl5B	−0.1718 (3)	0.55696 (6)	0.2089 (2)	0.0275 (6)	
C1B	−0.0147 (13)	0.6568 (2)	0.5751 (9)	0.0200 (19)	
C2B	−0.0462 (11)	0.62525 (19)	0.5291 (7)	0.0215 (19)	
H2B	−0.1336	0.6112	0.5523	0.026*	
C3B	0.0429 (12)	0.6137 (2)	0.4523 (7)	0.025 (2)	
H3B	0.0134	0.5926	0.4183	0.030*	
C4B	0.1814 (13)	0.6343 (2)	0.4246 (8)	0.0186 (19)	
C5B	0.2204 (12)	0.66517 (18)	0.4713 (7)	0.025 (2)	
H5B	0.3160	0.6785	0.4536	0.030*	
C6B	0.1199 (10)	0.67649 (19)	0.5436 (7)	0.0224 (19)	
H6B	0.1422	0.6983	0.5731	0.027*	
C7B	0.1908 (12)	0.56185 (19)	0.1911 (8)	0.0216 (10)	
H7B1	0.2934	0.5527	0.1608	0.026*	
H7B2	0.1566	0.5841	0.1580	0.026*	
C8B	0.0133 (13)	0.53908 (19)	0.1617 (8)	0.0214 (10)	
C1'B	−0.1193 (11)	0.66870 (19)	0.6604 (8)	0.0186 (10)	
C2'B	−0.0398 (11)	0.69443 (17)	0.7376 (6)	0.0211 (10)	
H2'B	0.0813	0.7036	0.7387	0.025*	
C3'B	−0.1394 (12)	0.70619 (17)	0.8115 (7)	0.0217 (10)	
C4'B	−0.3189 (11)	0.6936 (2)	0.8076 (7)	0.0230 (11)	
C5'B	−0.3956 (11)	0.66792 (19)	0.7326 (7)	0.0264 (11)	
H5'B	−0.5161	0.6586	0.7321	0.032*	
C6'B	−0.2966 (10)	0.65610 (18)	0.6594 (7)	0.0224 (11)	
H6'B	−0.3511	0.6390	0.6072	0.027*	
S1C	1.3915 (3)	0.58721 (5)	0.8798 (2)	0.0214 (6)	
O1C	1.2647 (9)	0.61955 (15)	0.8500 (5)	0.0242 (15)	
O2C	1.2418 (8)	0.55900 (14)	0.8285 (6)	0.0177 (13)	
O3C	1.4484 (9)	0.58174 (15)	0.9986 (6)	0.0258 (15)	
O4C	1.5231 (9)	0.58960 (16)	0.8136 (6)	0.0276 (16)	
Cl1C	0.9555 (3)	0.75553 (5)	1.35532 (17)	0.0298 (5)	
Cl2C	0.6170 (3)	0.71937 (5)	1.4213 (2)	0.0291 (6)	
Cl3C	1.0490 (3)	0.49568 (5)	0.7412 (2)	0.0263 (6)	
Cl4C	0.9415 (3)	0.52908 (6)	0.5218 (2)	0.0272 (6)	
Cl5C	0.8154 (3)	0.55435 (5)	0.7123 (2)	0.0234 (6)	
C1C	0.9952 (13)	0.6580 (2)	1.0860 (8)	0.0180 (18)	
C2C	0.8978 (12)	0.6360 (2)	1.0020 (7)	0.0213 (19)	
H2C	0.7686	0.6302	0.9967	0.026*	
C3C	0.9883 (11)	0.6224 (2)	0.9259 (8)	0.021 (2)	
H3C	0.9225	0.6070	0.8695	0.025*	
C4C	1.1745 (13)	0.6314 (2)	0.9331 (9)	0.020 (2)	
C5C	1.2746 (11)	0.65297 (18)	1.0156 (7)	0.0226 (19)	
H5C	1.4025	0.6591	1.0189	0.027*	
C6C	1.1847 (11)	0.66571 (18)	1.0941 (7)	0.0245 (19)	
H6C	1.2542	0.6798	1.1539	0.029*	
C7C	1.1868 (12)	0.55543 (19)	0.7048 (8)	0.0217 (10)	
H7C1	1.2900	0.5444	0.6795	0.026*	
H7C2	1.1613	0.5772	0.6671	0.026*	
C8C	1.0047 (12)	0.53442 (19)	0.6746 (8)	0.0180 (10)	
C1'C	0.9007 (11)	0.6729 (2)	1.1687 (8)	0.0194 (10)	
C2'C	0.9571 (11)	0.70446 (18)	1.2150 (7)	0.0220 (10)	
H2'C	1.0530	0.7163	1.1920	0.026*	
C3'C	0.8720 (11)	0.71828 (17)	1.2947 (7)	0.0191 (9)	
C4'C	0.7265 (10)	0.70184 (19)	1.3256 (7)	0.0207 (10)	
C5'C	0.6697 (10)	0.67109 (18)	1.2808 (7)	0.0239 (11)	
H5'C	0.5705	0.6597	1.3022	0.029*	
C6'C	0.7582 (10)	0.65668 (18)	1.2039 (6)	0.0222 (11)	
H6'C	0.7198	0.6353	1.1749	0.027*	
S1D	1.1226 (3)	0.40890 (5)	0.8723 (2)	0.0231 (6)	
O1D	1.2492 (9)	0.37714 (14)	0.8970 (6)	0.0249 (15)	
O2D	1.2695 (9)	0.43669 (15)	0.9271 (6)	0.0236 (14)	
O3D	1.0666 (9)	0.41554 (16)	0.7527 (6)	0.0258 (15)	
O4D	0.9884 (9)	0.40510 (15)	0.9376 (6)	0.0302 (17)	
Cl1D	1.5275 (3)	0.25787 (5)	0.33982 (19)	0.0384 (6)	
Cl2D	1.9395 (4)	0.28238 (6)	0.3372 (2)	0.0377 (6)	
Cl3D	1.4471 (3)	0.50056 (6)	1.0258 (2)	0.0277 (6)	
Cl4D	1.5717 (4)	0.46324 (6)	1.2378 (2)	0.0313 (6)	
Cl5D	1.6925 (3)	0.44383 (6)	1.0375 (2)	0.0271 (6)	
C1D	1.5371 (12)	0.3415 (2)	0.6623 (8)	0.0188 (19)	
C2D	1.5793 (11)	0.3726 (2)	0.7139 (7)	0.0188 (18)	
H2D	1.6748	0.3858	0.6954	0.023*	
C3D	1.4871 (12)	0.3845 (2)	0.7900 (7)	0.025 (2)	
H3D	1.5191	0.4056	0.8244	0.030*	
C4D	1.3491 (13)	0.3658 (2)	0.8158 (8)	0.020 (2)	
C5D	1.3035 (10)	0.33494 (18)	0.7725 (7)	0.0214 (18)	
H5D	1.2096	0.3221	0.7941	0.026*	
C6D	1.3979 (11)	0.32293 (19)	0.6966 (7)	0.0242 (19)	
H6D	1.3680	0.3014	0.6662	0.029*	
C7D	1.3283 (12)	0.43822 (19)	1.0533 (8)	0.0216 (10)	
H7D1	1.3613	0.4160	1.0866	0.026*	
H7D2	1.2240	0.4474	1.0820	0.026*	
C8D	1.5031 (13)	0.4606 (2)	1.0844 (8)	0.0214 (10)	
C1'D	1.6335 (11)	0.32815 (19)	0.5800 (8)	0.0186 (10)	
C2'D	1.5501 (11)	0.30334 (18)	0.5052 (6)	0.0211 (10)	
H2'D	1.4264	0.2956	0.5052	0.025*	
C3'D	1.6406 (12)	0.28921 (18)	0.4299 (7)	0.0217 (10)	
C4'D	1.8203 (11)	0.30051 (19)	0.4282 (7)	0.0230 (11)	
C5'D	1.9067 (11)	0.32561 (18)	0.5010 (7)	0.0264 (11)	
H5'D	2.0292	0.3336	0.4995	0.032*	
C6'D	1.8147 (10)	0.33916 (18)	0.5761 (6)	0.0224 (11)	
H6'D	1.8759	0.3563	0.6263	0.027*	

### Atomic displacement parameters (Å^2^)

**Table:**

	*U*^11^	*U*^22^	*U*^33^	*U*^12^	*U*^13^	*U*^23^
S1A	0.0144 (12)	0.0222 (12)	0.0243 (15)	0.0009 (9)	0.0012 (11)	−0.0047 (10)
O1A	0.022 (3)	0.018 (3)	0.019 (4)	0.003 (2)	0.005 (3)	−0.001 (2)
O2A	0.019 (3)	0.025 (3)	0.017 (3)	−0.004 (2)	−0.001 (3)	−0.003 (3)
O3A	0.022 (4)	0.033 (4)	0.019 (3)	0.009 (3)	−0.003 (3)	−0.007 (3)
O4A	0.016 (3)	0.037 (4)	0.035 (4)	−0.001 (3)	0.011 (3)	−0.014 (3)
Cl1A	0.0348 (13)	0.0293 (12)	0.0293 (13)	−0.0069 (9)	0.0126 (10)	−0.0087 (10)
Cl2A	0.0346 (13)	0.0305 (12)	0.0323 (13)	0.0078 (9)	0.0188 (11)	0.0069 (10)
Cl3A	0.0275 (13)	0.0194 (11)	0.0251 (14)	0.0000 (9)	0.0055 (10)	0.0018 (10)
Cl4A	0.0261 (13)	0.0303 (13)	0.0211 (13)	−0.0051 (9)	0.0055 (10)	−0.0025 (10)
Cl5A	0.0165 (12)	0.0257 (12)	0.0257 (15)	0.0004 (9)	0.0056 (11)	−0.0017 (10)
C1A	0.020 (5)	0.012 (4)	0.026 (5)	0.004 (3)	0.005 (4)	−0.001 (3)
C2A	0.015 (4)	0.023 (4)	0.028 (5)	−0.006 (3)	0.002 (4)	−0.001 (4)
C3A	0.032 (4)	0.013 (4)	0.025 (5)	−0.001 (3)	0.005 (4)	0.000 (3)
C4A	0.023 (5)	0.019 (4)	0.017 (5)	0.009 (3)	0.006 (4)	0.005 (3)
C5A	0.023 (4)	0.019 (4)	0.037 (5)	0.000 (3)	0.005 (4)	0.001 (4)
C6A	0.023 (4)	0.022 (4)	0.020 (5)	−0.004 (3)	0.000 (4)	0.000 (3)
C7A	0.022 (2)	0.021 (2)	0.020 (2)	−0.0052 (18)	0.0028 (19)	−0.0055 (19)
C8A	0.011 (2)	0.022 (2)	0.019 (2)	−0.0015 (17)	0.0019 (18)	−0.0006 (18)
C1'A	0.015 (2)	0.020 (2)	0.022 (3)	0.0018 (18)	0.0032 (19)	0.0026 (18)
C2'A	0.020 (2)	0.022 (2)	0.026 (3)	0.0031 (19)	0.009 (2)	0.0043 (19)
C3'A	0.021 (2)	0.015 (2)	0.018 (2)	−0.0054 (18)	0.0011 (18)	−0.0016 (18)
C4'A	0.020 (2)	0.017 (2)	0.028 (3)	0.0067 (18)	0.011 (2)	0.004 (2)
C5'A	0.020 (3)	0.020 (2)	0.033 (3)	0.0026 (19)	0.011 (2)	0.010 (2)
C6'A	0.023 (3)	0.017 (2)	0.025 (3)	−0.0019 (19)	0.004 (2)	0.002 (2)
S1B	0.0190 (13)	0.0205 (11)	0.0275 (15)	−0.0011 (9)	0.0034 (11)	−0.0024 (10)
O1B	0.023 (3)	0.021 (3)	0.019 (4)	0.001 (2)	0.004 (3)	0.000 (3)
O2B	0.023 (3)	0.019 (3)	0.014 (3)	−0.006 (2)	0.001 (3)	−0.001 (3)
O3B	0.030 (4)	0.027 (4)	0.024 (4)	0.001 (3)	−0.003 (3)	0.001 (3)
O4B	0.019 (3)	0.021 (3)	0.043 (4)	−0.006 (2)	0.012 (3)	−0.008 (3)
Cl1B	0.0393 (13)	0.0315 (12)	0.0332 (14)	−0.0022 (10)	0.0089 (11)	−0.0108 (10)
Cl2B	0.0449 (15)	0.0324 (12)	0.0309 (14)	0.0079 (10)	0.0208 (11)	0.0038 (11)
Cl3B	0.0262 (13)	0.0218 (11)	0.0287 (14)	−0.0015 (9)	0.0061 (10)	0.0000 (10)
Cl4B	0.0325 (14)	0.0342 (13)	0.0203 (13)	−0.0054 (10)	0.0024 (11)	−0.0041 (10)
Cl5B	0.0193 (13)	0.0335 (13)	0.0289 (16)	0.0020 (10)	0.0052 (11)	−0.0034 (11)
C1B	0.017 (4)	0.024 (4)	0.017 (5)	0.003 (3)	0.002 (4)	0.007 (3)
C2B	0.014 (4)	0.022 (4)	0.028 (5)	−0.004 (3)	0.006 (4)	0.000 (4)
C3B	0.036 (5)	0.017 (4)	0.021 (5)	−0.001 (3)	0.008 (4)	−0.001 (4)
C4B	0.022 (5)	0.018 (4)	0.017 (5)	0.006 (3)	0.007 (4)	0.003 (3)
C5B	0.033 (5)	0.018 (4)	0.026 (5)	−0.006 (3)	0.010 (4)	0.002 (4)
C6B	0.022 (5)	0.017 (4)	0.024 (5)	−0.003 (3)	0.001 (4)	−0.004 (4)
C7B	0.023 (3)	0.023 (2)	0.018 (2)	−0.0027 (19)	0.0032 (19)	0.0011 (19)
C8B	0.020 (3)	0.023 (2)	0.020 (2)	0.0023 (18)	0.004 (2)	−0.0017 (19)
C1'B	0.015 (2)	0.017 (2)	0.021 (3)	0.0037 (18)	0.0005 (18)	0.0009 (18)
C2'B	0.023 (2)	0.015 (2)	0.024 (3)	−0.0031 (18)	0.005 (2)	0.0013 (18)
C3'B	0.028 (3)	0.015 (2)	0.020 (3)	0.0018 (19)	0.005 (2)	0.0003 (19)
C4'B	0.026 (3)	0.025 (3)	0.018 (3)	0.009 (2)	0.006 (2)	0.005 (2)
C5'B	0.023 (3)	0.029 (3)	0.027 (3)	0.001 (2)	0.007 (2)	0.009 (2)
C6'B	0.021 (2)	0.021 (3)	0.022 (3)	−0.004 (2)	0.002 (2)	0.000 (2)
S1C	0.0168 (12)	0.0220 (12)	0.0247 (15)	−0.0005 (9)	0.0047 (11)	−0.0026 (10)
O1C	0.026 (3)	0.024 (3)	0.022 (4)	0.006 (2)	0.005 (3)	−0.002 (3)
O2C	0.015 (3)	0.020 (3)	0.017 (3)	0.000 (2)	0.004 (3)	0.001 (3)
O3C	0.024 (4)	0.024 (3)	0.023 (3)	0.000 (3)	−0.004 (3)	−0.004 (3)
O4C	0.019 (3)	0.034 (4)	0.034 (4)	−0.006 (3)	0.014 (3)	−0.004 (3)
Cl1C	0.0302 (12)	0.0263 (11)	0.0381 (14)	−0.0078 (9)	0.0179 (11)	−0.0122 (10)
Cl2C	0.0332 (13)	0.0271 (11)	0.0329 (14)	0.0021 (9)	0.0191 (11)	0.0018 (10)
Cl3C	0.0246 (13)	0.0213 (11)	0.0334 (15)	−0.0017 (9)	0.0086 (11)	−0.0008 (10)
Cl4C	0.0261 (13)	0.0366 (14)	0.0185 (13)	−0.0075 (10)	0.0055 (10)	−0.0055 (10)
Cl5C	0.0182 (13)	0.0247 (12)	0.0269 (15)	0.0012 (9)	0.0056 (11)	−0.0010 (11)
C1C	0.015 (4)	0.022 (4)	0.014 (5)	0.003 (3)	−0.001 (3)	0.007 (3)
C2C	0.015 (4)	0.024 (4)	0.023 (5)	−0.001 (3)	0.002 (4)	−0.001 (3)
C3C	0.010 (4)	0.023 (4)	0.030 (5)	−0.003 (3)	0.004 (4)	−0.007 (4)
C4C	0.021 (4)	0.016 (4)	0.026 (5)	−0.001 (3)	0.012 (4)	0.001 (3)
C5C	0.014 (4)	0.020 (4)	0.032 (5)	−0.003 (3)	0.003 (4)	−0.008 (4)
C6C	0.025 (4)	0.023 (4)	0.028 (5)	−0.001 (3)	0.012 (4)	−0.002 (4)
C7C	0.022 (2)	0.021 (2)	0.020 (2)	−0.0052 (18)	0.0028 (19)	−0.0055 (19)
C8C	0.011 (2)	0.022 (2)	0.019 (2)	−0.0015 (17)	0.0019 (18)	−0.0006 (18)
C1'C	0.015 (2)	0.020 (2)	0.022 (3)	0.0018 (18)	0.0032 (19)	0.0026 (18)
C2'C	0.020 (2)	0.022 (2)	0.026 (3)	0.0031 (19)	0.009 (2)	0.0043 (19)
C3'C	0.021 (2)	0.015 (2)	0.018 (2)	−0.0054 (18)	0.0011 (18)	−0.0016 (18)
C4'C	0.020 (2)	0.017 (2)	0.028 (3)	0.0067 (18)	0.011 (2)	0.004 (2)
C5'C	0.020 (3)	0.020 (2)	0.033 (3)	0.0026 (19)	0.011 (2)	0.010 (2)
C6'C	0.023 (3)	0.017 (2)	0.025 (3)	−0.0019 (19)	0.004 (2)	0.002 (2)
S1D	0.0183 (12)	0.0207 (11)	0.0268 (15)	−0.0013 (9)	0.0003 (11)	−0.0027 (10)
O1D	0.035 (4)	0.018 (3)	0.026 (4)	0.003 (2)	0.016 (3)	0.000 (3)
O2D	0.024 (3)	0.028 (3)	0.017 (3)	−0.003 (2)	0.002 (3)	−0.004 (3)
O3D	0.020 (3)	0.030 (4)	0.022 (3)	0.004 (3)	−0.002 (3)	0.000 (3)
O4D	0.025 (4)	0.033 (4)	0.035 (4)	−0.001 (3)	0.012 (3)	0.000 (3)
Cl1D	0.0458 (14)	0.0328 (13)	0.0382 (15)	−0.0103 (10)	0.0146 (12)	−0.0116 (11)
Cl2D	0.0480 (15)	0.0372 (13)	0.0348 (14)	0.0042 (11)	0.0231 (12)	0.0000 (11)
Cl3D	0.0303 (13)	0.0209 (11)	0.0291 (14)	0.0023 (9)	0.0038 (11)	−0.0007 (10)
Cl4D	0.0328 (14)	0.0374 (14)	0.0209 (14)	−0.0032 (10)	0.0030 (11)	−0.0035 (11)
Cl5D	0.0212 (13)	0.0306 (13)	0.0284 (16)	0.0038 (10)	0.0053 (12)	−0.0064 (11)
C1D	0.015 (4)	0.025 (4)	0.013 (5)	−0.001 (3)	−0.003 (3)	−0.002 (3)
C2D	0.013 (4)	0.029 (4)	0.012 (4)	−0.007 (3)	−0.001 (3)	0.000 (3)
C3D	0.037 (5)	0.014 (4)	0.024 (5)	−0.003 (3)	0.007 (4)	−0.003 (4)
C4D	0.019 (5)	0.027 (4)	0.011 (5)	0.001 (3)	0.002 (4)	0.000 (4)
C5D	0.016 (4)	0.023 (4)	0.024 (5)	0.008 (3)	0.003 (3)	0.005 (3)
C6D	0.029 (5)	0.017 (4)	0.028 (5)	−0.002 (3)	0.010 (4)	0.001 (4)
C7D	0.023 (3)	0.023 (2)	0.018 (2)	−0.0027 (19)	0.0032 (19)	0.0011 (19)
C8D	0.020 (3)	0.023 (2)	0.020 (2)	0.0023 (18)	0.004 (2)	−0.0017 (19)
C1'D	0.015 (2)	0.017 (2)	0.021 (3)	0.0037 (18)	0.0005 (18)	0.0009 (18)
C2'D	0.023 (2)	0.015 (2)	0.024 (3)	−0.0031 (18)	0.005 (2)	0.0013 (18)
C3'D	0.028 (3)	0.015 (2)	0.020 (3)	0.0018 (19)	0.005 (2)	0.0003 (19)
C4'D	0.026 (3)	0.025 (3)	0.018 (3)	0.009 (2)	0.006 (2)	0.005 (2)
C5'D	0.023 (3)	0.029 (3)	0.027 (3)	0.001 (2)	0.007 (2)	0.009 (2)
C6'D	0.021 (2)	0.021 (3)	0.022 (3)	−0.004 (2)	0.002 (2)	0.000 (2)

### Geometric parameters (Å, °)

**Table:**

S1A—O4A	1.425 (6)	S1C—O3C	1.399 (8)
S1A—O3A	1.428 (7)	S1C—O4C	1.410 (6)
S1A—O2A	1.564 (6)	S1C—O2C	1.581 (7)
S1A—O1A	1.571 (6)	S1C—O1C	1.586 (6)
O1A—C4A	1.431 (10)	O1C—C4C	1.427 (10)
O2A—C7A	1.459 (11)	O2C—C7C	1.447 (11)
Cl1A—C3'A	1.736 (7)	Cl1C—C3'C	1.716 (7)
Cl2A—C4'A	1.729 (8)	Cl2C—C4'C	1.730 (8)
Cl3A—C8A	1.767 (8)	Cl3C—C8C	1.756 (8)
Cl4A—C8A	1.765 (10)	Cl4C—C8C	1.792 (9)
Cl5A—C8A	1.780 (8)	Cl5C—C8C	1.759 (8)
C1A—C2A	1.401 (12)	C1C—C6C	1.385 (11)
C1A—C6A	1.406 (11)	C1C—C2C	1.391 (12)
C1A—C1'A	1.490 (11)	C1C—C1'C	1.490 (12)
C2A—C3A	1.385 (11)	C2C—C3C	1.385 (11)
C2A—H2A	0.9500	C2C—H2C	0.9500
C3A—C4A	1.360 (11)	C3C—C4C	1.378 (11)
C3A—H3A	0.9500	C3C—H3C	0.9500
C4A—C5A	1.389 (12)	C4C—C5C	1.375 (12)
C5A—C6A	1.358 (10)	C5C—C6C	1.394 (10)
C5A—H5A	0.9500	C5C—H5C	0.9500
C6A—H6A	0.9500	C6C—H6C	0.9500
C7A—C8A	1.527 (11)	C7C—C8C	1.528 (11)
C7A—H7A1	0.9900	C7C—H7C1	0.9900
C7A—H7A2	0.9900	C7C—H7C2	0.9900
C1'A—C2'A	1.372 (11)	C1'C—C6'C	1.388 (10)
C1'A—C6'A	1.400 (10)	C1'C—C2'C	1.413 (11)
C2'A—C3'A	1.370 (10)	C2'C—C3'C	1.399 (10)
C2'A—H2'A	0.9500	C2'C—H2'C	0.9500
C3'A—C4'A	1.407 (10)	C3'C—C4'C	1.385 (10)
C4'A—C5'A	1.368 (10)	C4'C—C5'C	1.378 (10)
C5'A—C6'A	1.387 (10)	C5'C—C6'C	1.399 (10)
C5'A—H5'A	0.9500	C5'C—H5'C	0.9500
C6'A—H6'A	0.9500	C6'C—H6'C	0.9500
S1B—O4B	1.403 (6)	S1D—O3D	1.417 (7)
S1B—O3B	1.411 (8)	S1D—O4D	1.423 (6)
S1B—O1B	1.584 (6)	S1D—O1D	1.563 (6)
S1B—O2B	1.589 (6)	S1D—O2D	1.567 (7)
O1B—C4B	1.426 (10)	O1D—C4D	1.449 (9)
O2B—C7B	1.449 (11)	O2D—C7D	1.469 (11)
Cl1B—C3'B	1.733 (8)	Cl1D—C3'D	1.727 (8)
Cl2B—C4'B	1.731 (8)	Cl2D—C4'D	1.741 (8)
Cl3B—C8B	1.750 (9)	Cl3D—C8D	1.774 (8)
Cl4B—C8B	1.782 (10)	Cl4D—C8D	1.788 (10)
Cl5B—C8B	1.756 (9)	Cl5D—C8D	1.761 (9)
C1B—C2B	1.392 (12)	C1D—C2D	1.407 (11)
C1B—C6B	1.393 (11)	C1D—C6D	1.411 (11)
C1B—C1'B	1.520 (12)	C1D—C1'D	1.471 (11)
C2B—C3B	1.354 (10)	C2D—C3D	1.368 (11)
C2B—H2B	0.9500	C2D—H2D	0.9500
C3B—C4B	1.416 (11)	C3D—C4D	1.360 (11)
C3B—H3B	0.9500	C3D—H3D	0.9500
C4B—C5B	1.372 (11)	C4D—C5D	1.363 (11)
C5B—C6B	1.365 (10)	C5D—C6D	1.380 (10)
C5B—H5B	0.9500	C5D—H5D	0.9500
C6B—H6B	0.9500	C6D—H6D	0.9500
C7B—C8B	1.543 (12)	C7D—C8D	1.518 (12)
C7B—H7B1	0.9900	C7D—H7D1	0.9900
C7B—H7B2	0.9900	C7D—H7D2	0.9900
C1'B—C6'B	1.381 (10)	C1'D—C2'D	1.376 (11)
C1'B—C2'B	1.413 (11)	C1'D—C6'D	1.401 (10)
C2'B—C3'B	1.382 (10)	C2'D—C3'D	1.388 (10)
C2'B—H2'B	0.9500	C2'D—H2'D	0.9500
C3'B—C4'B	1.387 (10)	C3'D—C4'D	1.387 (10)
C4'B—C5'B	1.393 (10)	C4'D—C5'D	1.379 (11)
C5'B—C6'B	1.373 (10)	C5'D—C6'D	1.383 (10)
C5'B—H5'B	0.9500	C5'D—H5'D	0.9500
C6'B—H6'B	0.9500	C6'D—H6'D	0.9500
			
O4A—S1A—O3A	122.5 (4)	O3C—S1C—O4C	122.9 (4)
O4A—S1A—O2A	109.4 (4)	O3C—S1C—O2C	105.2 (4)
O3A—S1A—O2A	105.1 (4)	O4C—S1C—O2C	109.0 (4)
O4A—S1A—O1A	104.9 (4)	O3C—S1C—O1C	110.6 (4)
O3A—S1A—O1A	109.7 (4)	O4C—S1C—O1C	104.7 (4)
O2A—S1A—O1A	103.9 (3)	O2C—S1C—O1C	102.7 (3)
C4A—O1A—S1A	118.0 (6)	C4C—O1C—S1C	117.9 (6)
C7A—O2A—S1A	116.1 (5)	C7C—O2C—S1C	116.2 (5)
C2A—C1A—C6A	116.9 (8)	C6C—C1C—C2C	119.2 (8)
C2A—C1A—C1'A	121.8 (7)	C6C—C1C—C1'C	119.5 (8)
C6A—C1A—C1'A	121.3 (8)	C2C—C1C—C1'C	121.2 (7)
C3A—C2A—C1A	121.3 (8)	C3C—C2C—C1C	120.3 (8)
C3A—C2A—H2A	119.3	C3C—C2C—H2C	119.8
C1A—C2A—H2A	119.3	C1C—C2C—H2C	119.8
C4A—C3A—C2A	118.8 (8)	C4C—C3C—C2C	119.3 (8)
C4A—C3A—H3A	120.6	C4C—C3C—H3C	120.3
C2A—C3A—H3A	120.6	C2C—C3C—H3C	120.3
C3A—C4A—C5A	122.4 (8)	C5C—C4C—C3C	121.6 (8)
C3A—C4A—O1A	120.2 (8)	C5C—C4C—O1C	118.3 (7)
C5A—C4A—O1A	117.3 (8)	C3C—C4C—O1C	120.0 (8)
C6A—C5A—C4A	118.1 (8)	C4C—C5C—C6C	118.7 (7)
C6A—C5A—H5A	121.0	C4C—C5C—H5C	120.6
C4A—C5A—H5A	121.0	C6C—C5C—H5C	120.6
C5A—C6A—C1A	122.5 (8)	C1C—C6C—C5C	120.7 (8)
C5A—C6A—H6A	118.7	C1C—C6C—H6C	119.7
C1A—C6A—H6A	118.7	C5C—C6C—H6C	119.7
O2A—C7A—C8A	107.0 (7)	O2C—C7C—C8C	106.0 (7)
O2A—C7A—H7A1	110.3	O2C—C7C—H7C1	110.5
C8A—C7A—H7A1	110.3	C8C—C7C—H7C1	110.5
O2A—C7A—H7A2	110.3	O2C—C7C—H7C2	110.5
C8A—C7A—H7A2	110.3	C8C—C7C—H7C2	110.5
H7A1—C7A—H7A2	108.6	H7C1—C7C—H7C2	108.7
C7A—C8A—Cl4A	106.6 (6)	C7C—C8C—Cl3C	110.8 (6)
C7A—C8A—Cl3A	111.0 (6)	C7C—C8C—Cl5C	111.4 (6)
Cl4A—C8A—Cl3A	109.9 (4)	Cl3C—C8C—Cl5C	110.4 (5)
C7A—C8A—Cl5A	110.7 (5)	C7C—C8C—Cl4C	105.7 (6)
Cl4A—C8A—Cl5A	109.7 (5)	Cl3C—C8C—Cl4C	109.1 (4)
Cl3A—C8A—Cl5A	108.9 (5)	Cl5C—C8C—Cl4C	109.3 (5)
C2'A—C1'A—C6'A	119.2 (7)	C6'C—C1'C—C2'C	117.7 (7)
C2'A—C1'A—C1A	122.5 (7)	C6'C—C1'C—C1C	122.4 (7)
C6'A—C1'A—C1A	118.3 (7)	C2'C—C1'C—C1C	119.9 (7)
C3'A—C2'A—C1'A	121.4 (7)	C3'C—C2'C—C1'C	120.2 (7)
C3'A—C2'A—H2'A	119.3	C3'C—C2'C—H2'C	119.9
C1'A—C2'A—H2'A	119.3	C1'C—C2'C—H2'C	119.9
C2'A—C3'A—C4'A	120.0 (7)	C4'C—C3'C—C2'C	120.5 (7)
C2'A—C3'A—Cl1A	120.2 (6)	C4'C—C3'C—Cl1C	120.9 (6)
C4'A—C3'A—Cl1A	119.8 (6)	C2'C—C3'C—Cl1C	118.5 (6)
C5'A—C4'A—C3'A	118.7 (7)	C5'C—C4'C—C3'C	119.8 (7)
C5'A—C4'A—Cl2A	120.5 (6)	C5'C—C4'C—Cl2C	119.6 (6)
C3'A—C4'A—Cl2A	120.8 (6)	C3'C—C4'C—Cl2C	120.6 (6)
C4'A—C5'A—C6'A	121.4 (7)	C4'C—C5'C—C6'C	119.9 (7)
C4'A—C5'A—H5'A	119.3	C4'C—C5'C—H5'C	120.0
C6'A—C5'A—H5'A	119.3	C6'C—C5'C—H5'C	120.0
C5'A—C6'A—C1'A	119.3 (7)	C1'C—C6'C—C5'C	121.7 (7)
C5'A—C6'A—H6'A	120.4	C1'C—C6'C—H6'C	119.2
C1'A—C6'A—H6'A	120.4	C5'C—C6'C—H6'C	119.2
O4B—S1B—O3B	122.1 (4)	O3D—S1D—O4D	122.9 (4)
O4B—S1B—O1B	104.6 (4)	O3D—S1D—O1D	109.8 (4)
O3B—S1B—O1B	110.8 (4)	O4D—S1D—O1D	105.1 (4)
O4B—S1B—O2B	109.8 (4)	O3D—S1D—O2D	105.3 (4)
O3B—S1B—O2B	105.1 (4)	O4D—S1D—O2D	109.3 (4)
O1B—S1B—O2B	103.1 (3)	O1D—S1D—O2D	102.7 (4)
C4B—O1B—S1B	119.8 (6)	C4D—O1D—S1D	120.7 (6)
C7B—O2B—S1B	115.8 (5)	C7D—O2D—S1D	116.0 (5)
C2B—C1B—C6B	117.8 (8)	C2D—C1D—C6D	115.7 (7)
C2B—C1B—C1'B	120.5 (7)	C2D—C1D—C1'D	123.1 (7)
C6B—C1B—C1'B	121.7 (8)	C6D—C1D—C1'D	121.1 (7)
C3B—C2B—C1B	122.5 (8)	C3D—C2D—C1D	122.0 (8)
C3B—C2B—H2B	118.8	C3D—C2D—H2D	119.0
C1B—C2B—H2B	118.8	C1D—C2D—H2D	119.0
C2B—C3B—C4B	117.7 (8)	C4D—C3D—C2D	119.1 (8)
C2B—C3B—H3B	121.2	C4D—C3D—H3D	120.5
C4B—C3B—H3B	121.2	C2D—C3D—H3D	120.5
C5B—C4B—C3B	121.2 (8)	C3D—C4D—C5D	122.7 (8)
C5B—C4B—O1B	117.5 (8)	C3D—C4D—O1D	121.1 (8)
C3B—C4B—O1B	121.2 (8)	C5D—C4D—O1D	116.1 (7)
C6B—C5B—C4B	119.1 (8)	C4D—C5D—C6D	118.0 (8)
C6B—C5B—H5B	120.4	C4D—C5D—H5D	121.0
C4B—C5B—H5B	120.4	C6D—C5D—H5D	121.0
C5B—C6B—C1B	121.5 (8)	C5D—C6D—C1D	122.4 (7)
C5B—C6B—H6B	119.2	C5D—C6D—H6D	118.8
C1B—C6B—H6B	119.2	C1D—C6D—H6D	118.8
O2B—C7B—C8B	105.3 (7)	O2D—C7D—C8D	105.4 (7)
O2B—C7B—H7B1	110.7	O2D—C7D—H7D1	110.7
C8B—C7B—H7B1	110.7	C8D—C7D—H7D1	110.7
O2B—C7B—H7B2	110.7	O2D—C7D—H7D2	110.7
C8B—C7B—H7B2	110.7	C8D—C7D—H7D2	110.7
H7B1—C7B—H7B2	108.8	H7D1—C7D—H7D2	108.8
C7B—C8B—Cl3B	111.1 (6)	C7D—C8D—Cl5D	111.2 (6)
C7B—C8B—Cl5B	109.9 (6)	C7D—C8D—Cl3D	111.4 (6)
Cl3B—C8B—Cl5B	110.6 (5)	Cl5D—C8D—Cl3D	109.6 (5)
C7B—C8B—Cl4B	105.2 (6)	C7D—C8D—Cl4D	105.4 (6)
Cl3B—C8B—Cl4B	110.1 (5)	Cl5D—C8D—Cl4D	110.0 (5)
Cl5B—C8B—Cl4B	109.8 (5)	Cl3D—C8D—Cl4D	109.1 (5)
C6'B—C1'B—C2'B	119.1 (7)	C2'D—C1'D—C6'D	117.1 (7)
C6'B—C1'B—C1B	121.1 (7)	C2'D—C1'D—C1D	120.7 (7)
C2'B—C1'B—C1B	119.7 (7)	C6'D—C1'D—C1D	122.2 (7)
C3'B—C2'B—C1'B	119.6 (7)	C1'D—C2'D—C3'D	122.3 (7)
C3'B—C2'B—H2'B	120.2	C1'D—C2'D—H2'D	118.9
C1'B—C2'B—H2'B	120.2	C3'D—C2'D—H2'D	118.9
C2'B—C3'B—C4'B	120.3 (7)	C4'D—C3'D—C2'D	119.4 (7)
C2'B—C3'B—Cl1B	118.5 (6)	C4'D—C3'D—Cl1D	121.1 (6)
C4'B—C3'B—Cl1B	121.1 (6)	C2'D—C3'D—Cl1D	119.5 (6)
C3'B—C4'B—C5'B	119.9 (7)	C5'D—C4'D—C3'D	119.8 (7)
C3'B—C4'B—Cl2B	120.3 (6)	C5'D—C4'D—Cl2D	120.0 (6)
C5'B—C4'B—Cl2B	119.7 (6)	C3'D—C4'D—Cl2D	120.2 (6)
C6'B—C5'B—C4'B	119.8 (7)	C4'D—C5'D—C6'D	119.9 (7)
C6'B—C5'B—H5'B	120.1	C4'D—C5'D—H5'D	120.1
C4'B—C5'B—H5'B	120.1	C6'D—C5'D—H5'D	120.1
C5'B—C6'B—C1'B	121.2 (7)	C5'D—C6'D—C1'D	121.6 (7)
C5'B—C6'B—H6'B	119.4	C5'D—C6'D—H6'D	119.2
C1'B—C6'B—H6'B	119.4	C1'D—C6'D—H6'D	119.2
			
O4A—S1A—O1A—C4A	158.0 (6)	O3C—S1C—O1C—C4C	−20.6 (7)
O3A—S1A—O1A—C4A	24.7 (7)	O4C—S1C—O1C—C4C	−154.9 (6)
O2A—S1A—O1A—C4A	−87.2 (7)	O2C—S1C—O1C—C4C	91.3 (7)
O4A—S1A—O2A—C7A	36.5 (7)	O3C—S1C—O2C—C7C	−169.1 (6)
O3A—S1A—O2A—C7A	169.7 (6)	O4C—S1C—O2C—C7C	−35.6 (7)
O1A—S1A—O2A—C7A	−75.1 (6)	O1C—S1C—O2C—C7C	75.1 (6)
C6A—C1A—C2A—C3A	−1.5 (12)	C6C—C1C—C2C—C3C	−1.3 (13)
C1'A—C1A—C2A—C3A	−179.5 (8)	C1'C—C1C—C2C—C3C	−179.9 (8)
C1A—C2A—C3A—C4A	−0.2 (12)	C1C—C2C—C3C—C4C	−1.1 (13)
C2A—C3A—C4A—C5A	1.2 (13)	C2C—C3C—C4C—C5C	1.4 (14)
C2A—C3A—C4A—O1A	177.4 (7)	C2C—C3C—C4C—O1C	−175.2 (8)
S1A—O1A—C4A—C3A	91.2 (9)	S1C—O1C—C4C—C5C	89.0 (9)
S1A—O1A—C4A—C5A	−92.4 (8)	S1C—O1C—C4C—C3C	−94.3 (9)
C3A—C4A—C5A—C6A	−0.5 (13)	C3C—C4C—C5C—C6C	0.7 (14)
O1A—C4A—C5A—C6A	−176.8 (7)	O1C—C4C—C5C—C6C	177.4 (7)
C4A—C5A—C6A—C1A	−1.3 (13)	C2C—C1C—C6C—C5C	3.4 (13)
C2A—C1A—C6A—C5A	2.2 (13)	C1'C—C1C—C6C—C5C	−177.9 (8)
C1'A—C1A—C6A—C5A	−179.7 (8)	C4C—C5C—C6C—C1C	−3.1 (13)
S1A—O2A—C7A—C8A	167.0 (5)	S1C—O2C—C7C—C8C	−164.1 (5)
O2A—C7A—C8A—Cl4A	177.8 (5)	O2C—C7C—C8C—Cl3C	−60.2 (7)
O2A—C7A—C8A—Cl3A	58.1 (7)	O2C—C7C—C8C—Cl5C	63.1 (8)
O2A—C7A—C8A—Cl5A	−62.9 (7)	O2C—C7C—C8C—Cl4C	−178.3 (5)
C2A—C1A—C1'A—C2'A	152.5 (8)	C6C—C1C—C1'C—C6'C	−148.8 (8)
C6A—C1A—C1'A—C2'A	−25.4 (13)	C2C—C1C—C1'C—C6'C	29.8 (13)
C2A—C1A—C1'A—C6'A	−29.5 (12)	C6C—C1C—C1'C—C2'C	30.5 (12)
C6A—C1A—C1'A—C6'A	152.5 (8)	C2C—C1C—C1'C—C2'C	−150.9 (8)
C6'A—C1'A—C2'A—C3'A	0.2 (12)	C6'C—C1'C—C2'C—C3'C	0.8 (12)
C1A—C1'A—C2'A—C3'A	178.2 (8)	C1C—C1'C—C2'C—C3'C	−178.6 (8)
C1'A—C2'A—C3'A—C4'A	1.1 (12)	C1'C—C2'C—C3'C—C4'C	−2.6 (12)
C1'A—C2'A—C3'A—Cl1A	−175.7 (7)	C1'C—C2'C—C3'C—Cl1C	176.0 (6)
C2'A—C3'A—C4'A—C5'A	−0.5 (12)	C2'C—C3'C—C4'C—C5'C	2.4 (12)
Cl1A—C3'A—C4'A—C5'A	176.3 (6)	Cl1C—C3'C—C4'C—C5'C	−176.2 (6)
C2'A—C3'A—C4'A—Cl2A	177.9 (6)	C2'C—C3'C—C4'C—Cl2C	−178.3 (6)
Cl1A—C3'A—C4'A—Cl2A	−5.3 (10)	Cl1C—C3'C—C4'C—Cl2C	3.0 (10)
C3'A—C4'A—C5'A—C6'A	−1.5 (12)	C3'C—C4'C—C5'C—C6'C	−0.4 (12)
Cl2A—C4'A—C5'A—C6'A	−179.9 (6)	Cl2C—C4'C—C5'C—C6'C	−179.7 (6)
C4'A—C5'A—C6'A—C1'A	2.9 (12)	C2'C—C1'C—C6'C—C5'C	1.2 (12)
C2'A—C1'A—C6'A—C5'A	−2.2 (12)	C1C—C1'C—C6'C—C5'C	−179.4 (8)
C1A—C1'A—C6'A—C5'A	179.8 (8)	C4'C—C5'C—C6'C—C1'C	−1.4 (12)
O4B—S1B—O1B—C4B	−159.3 (7)	O3D—S1D—O1D—C4D	21.9 (8)
O3B—S1B—O1B—C4B	−26.0 (8)	O4D—S1D—O1D—C4D	155.9 (7)
O2B—S1B—O1B—C4B	85.9 (7)	O2D—S1D—O1D—C4D	−89.7 (7)
O4B—S1B—O2B—C7B	−37.4 (7)	O3D—S1D—O2D—C7D	171.3 (6)
O3B—S1B—O2B—C7B	−170.4 (6)	O4D—S1D—O2D—C7D	37.4 (7)
O1B—S1B—O2B—C7B	73.5 (6)	O1D—S1D—O2D—C7D	−73.8 (6)
C6B—C1B—C2B—C3B	−2.6 (13)	C6D—C1D—C2D—C3D	−1.8 (12)
C1'B—C1B—C2B—C3B	179.6 (8)	C1'D—C1D—C2D—C3D	179.8 (8)
C1B—C2B—C3B—C4B	4.0 (13)	C1D—C2D—C3D—C4D	−0.6 (13)
C2B—C3B—C4B—C5B	−1.8 (13)	C2D—C3D—C4D—C5D	2.8 (14)
C2B—C3B—C4B—O1B	179.5 (7)	C2D—C3D—C4D—O1D	179.2 (7)
S1B—O1B—C4B—C5B	123.2 (8)	S1D—O1D—C4D—C3D	62.7 (11)
S1B—O1B—C4B—C3B	−58.1 (11)	S1D—O1D—C4D—C5D	−120.7 (8)
C3B—C4B—C5B—C6B	−1.6 (13)	C3D—C4D—C5D—C6D	−2.3 (14)
O1B—C4B—C5B—C6B	177.1 (8)	O1D—C4D—C5D—C6D	−178.8 (7)
C4B—C5B—C6B—C1B	3.0 (13)	C4D—C5D—C6D—C1D	−0.4 (12)
C2B—C1B—C6B—C5B	−1.0 (13)	C2D—C1D—C6D—C5D	2.4 (12)
C1'B—C1B—C6B—C5B	176.8 (8)	C1'D—C1D—C6D—C5D	−179.2 (8)
S1B—O2B—C7B—C8B	−167.3 (5)	S1D—O2D—C7D—C8D	166.1 (5)
O2B—C7B—C8B—Cl3B	−59.0 (7)	O2D—C7D—C8D—Cl5D	−62.1 (7)
O2B—C7B—C8B—Cl5B	63.8 (7)	O2D—C7D—C8D—Cl3D	60.5 (7)
O2B—C7B—C8B—Cl4B	−178.1 (5)	O2D—C7D—C8D—Cl4D	178.7 (5)
C2B—C1B—C1'B—C6'B	−27.4 (13)	C2D—C1D—C1'D—C2'D	−159.1 (8)
C6B—C1B—C1'B—C6'B	154.9 (8)	C6D—C1D—C1'D—C2'D	22.6 (13)
C2B—C1B—C1'B—C2'B	156.4 (8)	C2D—C1D—C1'D—C6'D	22.8 (13)
C6B—C1B—C1'B—C2'B	−21.3 (13)	C6D—C1D—C1'D—C6'D	−155.4 (8)
C6'B—C1'B—C2'B—C3'B	0.8 (12)	C6'D—C1'D—C2'D—C3'D	0.9 (12)
C1B—C1'B—C2'B—C3'B	177.1 (7)	C1D—C1'D—C2'D—C3'D	−177.2 (8)
C1'B—C2'B—C3'B—C4'B	−2.0 (12)	C1'D—C2'D—C3'D—C4'D	−0.7 (12)
C1'B—C2'B—C3'B—Cl1B	180.0 (6)	C1'D—C2'D—C3'D—Cl1D	179.3 (6)
C2'B—C3'B—C4'B—C5'B	2.9 (12)	C2'D—C3'D—C4'D—C5'D	−0.1 (12)
Cl1B—C3'B—C4'B—C5'B	−179.1 (6)	Cl1D—C3'D—C4'D—C5'D	179.9 (6)
C2'B—C3'B—C4'B—Cl2B	179.1 (6)	C2'D—C3'D—C4'D—Cl2D	178.2 (6)
Cl1B—C3'B—C4'B—Cl2B	−2.9 (10)	Cl1D—C3'D—C4'D—Cl2D	−1.8 (10)
C3'B—C4'B—C5'B—C6'B	−2.7 (12)	C3'D—C4'D—C5'D—C6'D	0.7 (12)
Cl2B—C4'B—C5'B—C6'B	−178.9 (6)	Cl2D—C4'D—C5'D—C6'D	−177.7 (6)
C4'B—C5'B—C6'B—C1'B	1.5 (12)	C4'D—C5'D—C6'D—C1'D	−0.4 (12)
C2'B—C1'B—C6'B—C5'B	−0.6 (12)	C2'D—C1'D—C6'D—C5'D	−0.3 (12)
C1B—C1'B—C6'B—C5'B	−176.8 (8)	C1D—C1'D—C6'D—C5'D	177.7 (8)
